# The personalization of engagement: the symbolic construction of social media and grassroots mobilization in Canadian newspapers

**DOI:** 10.1177/0163443717734406

**Published:** 2017-10-25

**Authors:** Delia Dumitrica, Maria Bakardjieva

**Affiliations:** Erasmus University, The Netherlands; University of Calgary, Canada

**Keywords:** Canada, civic action, civic engagement, discursive articulation, grassroots mobilization, media coverage, qualitative content analysis, social media

## Abstract

This article explores the symbolic construction of civic engagement mediated by social media in Canadian newspapers. The integration of social media in politics has created a discursive opening for reimagining engagement, partly as a result of enthusiastic accounts of the impact of digital technologies upon democracy. By means of a qualitative content analysis of Canadian newspaper articles between 2005 and 2014, we identify several discursive articulations of engagement: First, the articles offer the picture of a wide range of objects of engagement, suggesting a civic body actively involved in governance processes. Second, engagement appears to take place only reactively, after decisions are made. Finally, social media become the new social glue, bringing isolated individuals together and thus enabling them to pressure decision-making institutions. We argue that, collectively, these stories construct engagement as a deeply personal gesture that is nevertheless turned into a communal experience by the affordances of technology. The conclusion unpacks what we deem as the ambiguity at the heart of this discourse, considering its implications for democratic politics and suggesting avenues for the further monitoring of the technologically enabled personalization of engagement.

## Introduction

Democratic politics rests upon the idea of civic engagement. Traditionally, some types of engagement – voting, contacting elected officials, joining a political party – have been regarded as more important than others; but the forms of civic action that a society deems legitimate and effective change with time (Tilly, 1985). Furthermore, attempts to define and classify forms of engagement are themselves informed by specific understandings of democracy, involving exclusions that only legitimize particular forms of action over others (Cairns and Sears, 2012; Ekman and Amnå, 2012). Thus, engagement and civic action are contested political and discursive terrains, whose articulation legitimizes particular visions of democratic politics.

The integration of social media in politics has been accompanied by new stock phrases and narratives about what citizens *can* and *should* do to have a say in political matters. Is the discursive construction of engagement – and implicitly of democratic politics – changing with the growing popularity of social media, and if so, in what direction? Undoubtedly, discourses of digitally mediated engagement have been influenced by the optimistic rhetorical speculation concerning the alleged impact of technology on social life. ‘[F]ramed in the perspective of a *total revolution*, which means a democratic revolution in politics and public governance, or of a *technological fix* for basic problems of political activity and the trust of citizens in government’ (Van Dijk, 2012: 50), this optimism was rehearsed in the media coverage of some of the most prominent contemporary waves of resistance to existing political and economic structures (e.g. 15M, Indignados, Occupy, the Arab Spring, etc.) and further encapsulated in metaphors such as the ‘Twitter Revolution’ or the ‘social media revolution’. These metaphors have come to refer to any form of mass mobilization that uses social media as part of its communication repertoire, transforming these technologies into signifiers of citizen power – that is, the power of citizens to take part in the governance of their lives.

In this article, we inquire into the structure of these discourses by examining the Canadian newspapers’ symbolic construction of civic engagement mediated by social media such as Facebook, Twitter, or YouTube. We take the signifying work performed by newspapers as both reflecting and producing the contemporary civic culture, understood as ‘those features of the socio-cultural world – dispositions, practices, processes – that constitute the pre-conditions for people’s actual participation in the public sphere, in civil and political society’ (Dahlgren, 2009: 103). Our exploratory study maps how the processes of ‘becoming’ (or the transformation of individuals into) citizens are represented in Canadian print media. This allows us to consider the potential implications of this kind of symbolic construction of engagement for democratic politics. We show that stories about social media enabled engagement produce an image of an active citizenry, constantly monitoring power structures. This view contrasts starkly with recurring worries over political apathy and disengagement. Furthermore, this discursive articulation of technologically mediated engagement remains profoundly ambiguous: while it encourages citizens to imagine themselves as active political actors, it also casts engagement as a deeply personal gesture that does not require an (ethical) engagement with the Other.

While the Canadian case cannot be seen as representative of other Western societies, it is nevertheless indicative of developments unfolding in other liberal-democratic settings with high rates of social media adoption. Canada is at the forefront of social media integration not only in the daily practices of citizens (InsightsWest, 2016), but also in political communication (Dumitrica, 2014; Marland et al., 2014; Reilly, 2011; Small, 2011; Taras, 2015) and social movements (Callison and Hermida, 2015; Raynauld et al., 2017). This normalization of social media as political spaces and tools makes the Canadian case suitable for exploring the implications of the discursive construction of digitally mediated engagement for democratic politics more broadly. Furthermore, given the paucity of empirical research on media representations of technologically mediated engagement, our exploratory contribution develops a preliminary mapping of this problematic that can be further examined in other empirical studies and theoretical reflections beyond the Canadian context.

The article starts with an overview of the role of mass media in the symbolic construction of engagement. After dealing with the knotty question of the definition of engagement, this section reviews media’s contribution to the cultural understanding of civic action. The discussion here draws from the protest paradigm literature, identifying a need for empirical research into how civic engagement becomes constructed in mass media coverage. After a brief discussion of the methodological framework underpinning this project, the article moves on to presenting the findings by focusing on three elements of the coverage of technologically facilitated instances of engagement: the issues at stake, the drivers of the civic action, and the roles assigned to social media. We argue that, collectively, these stories construct engagement as a deeply personal gesture that is nevertheless turned into a communal experience by the affordances of technology. The conclusion unpacks what we deem as the ambiguity at the heart of this discourse, considering its implications for democratic politics and suggesting avenues for the further monitoring of the technologically enabled personalization of engagement.

## Engagement and media representations

Etymologically, engagement means to involve yourself into something. But when it comes to politics, not all forms of involvement are seen as equal. In the field of political communication, engagement has traditionally meant participation in electoral activities and contacting public officials (Verba, Nie, and Kim, in Ekman and Amnå, 2012: 286).^1^ Such definitions have been reluctantly expanded to account for less formal activities, where dispersed individuals seek common goals but lack ‘established political institutions to engage in public action towards these goals’ (McFarland, 2010: 9). Mundane communication and action are the ‘pre-political’ domain where citizens work out their positions by means of interaction with others (Dahlgren, 2013). Defining engagement, its ‘legitimate’ and ‘worthy’ forms, is thus an important dimension of imagining democratic politics (Cairns and Sears, 2012). Along with other meaning-making institutions, mass media are part and parcel of this process, providing and circulating vocabularies, arguments, and myths from which individuals draw in making sense of politics and their own role in it (Gamson, 1992).

### The protest paradigm

Civic cultures can facilitate or hinder ‘people acting as political agents’ (Dahlgren, 2013: 24). To what extent do mass media circulate discursive articulations enabling individuals to understand themselves as such political agents? An important body of literature in the field of communication attempting to shed light on this matter has focused on media coverage of protests (Cottle, 2008). This is not surprising, as protest has become a symbol of civic action (Van Aelst and Walgrave, 2001; see also McCurdy, 2012).

Two major studies of media in the 1960s (Gitlin, 1980; Halloran et al., 1970) gave rise to the protest paradigm literature (Chan and Lee, 1984), suggesting that media portrayals trivialize and delegitimize contentious civic action (Cottle, 2008). This is achieved through two persistent forms of bias: selection (which protests get coverage) and description (how protests are depicted; McCarthy et al., 1996). News stories often present protesters as deviants or disruptors of order, as reporting focuses on violence, conflict, and the spectacular dimension of civic action as opposed to the reasons for or the structural inequalities behind it.

While media institutions often espouse different editorial and ideological positions, potentially leading to different representations of engagement (e.g. Milne, 2005; Weaver and Scacco, 2013), recent studies largely confirm the continuation of the protest paradigm (e.g. Brasted, 2005; DeLuca et al., 2012). Unfortunately, there is little scholarly attention to Canadian media coverage of protest; the few published studies suggest that the protest paradigm is present, albeit certainly not uniformly reproduced across all media institutions (Corrigall-Brown and Wilkes, 2012; Gulliver and Herriot, 2015; Wilkes et al., 2010; Wittebols, 1996).

### Reporting protest in the time of social media

Internet challenges to mass media cannot be simplistically reduced to a matter of new media replacing legacy media. Media are best described as a hybrid system, ‘built upon interactions among older and newer media logics’ (Chadwick, 2013: 4). Because of our interest in those interactions that may affect the reporting of protest, next we look at the rise of online alternative media and civic journalism. These terms refer to the production of media texts by amateurs and/or activists outside traditional media institutions, a process praised as the democratization of news making (Allan, 2013; Kang, 2016). Amateur/activist participation in news making can take multiple forms, from uploading videos documenting events as they unfold to political commentary and analysis via blogging. Against ongoing scholarly concerns with the bias of media representation, citizen journalism promises to give voice to different positions, perspectives, and issues. For our purposes here, this suggests that, as traditional mass media are no longer and not necessarily controlling the framing of contentious civic action, the protest paradigm may be shifting. Alternative coverage of civic action can be provided (e.g. via blogs or Twitter) by citizen and activist sources, enabling participants to produce and circulate their own self-representation (Poell, 2014; Poell and Borra, 2011).

While the medium may be different, mediation remains nonetheless shaped by the political and economic structures within which it takes place (Cammaerts, 2012). In the remainder of this section, we explore several reasons for a skeptical position on the impact (at least in the short term) of citizen journalism on the protest paradigm or the symbolic construction of engagement.

First, the relationship between citizen-produced content and mainstream media is complex. Journalists and newsrooms are often skeptical of online material that cannot be independently verified. Furthermore, the (technical) quality of the citizen-produced content may not be good enough for use or may contravene professional guidelines (e.g. explicit violence). This may, in practice, limit opportunities for such content to make it into traditional media (Broersma and Graham, 2012; Hermida and Thurman, 2008; Hladík and Štětka, 2015; Knight, 2012; Mare, 2013; Paulussen and Harder, 2014; Van Leuven et al., 2015; Vis, 2013). As citizen journalists, alternative media and journalists, and newsrooms learn about each other’s expectations, their practices may eventually converge (Hänska and Shapour, 2013). Yet, given the asymmetric power relations between them, this is more likely to entail the adaptation of user-generated or alternative media content to the logic of traditional media (Lester and Hutchins, 2009). This is even more problematic in the context of activism’s adaptation to the social media logic, which may exacerbate the symbolic construction of protest as a (short-term) spectacle (e.g. as citizens document violence), potentially perpetuating the trivialization of protest that is central to the protest paradigm thesis (Poell, 2014; Poell and Borra, 2011). This results in a paradoxical situation, where civic organizers try to control their own representations in the media by internalizing the requirement for news to capture the spectacular (Bishop, 2013).

Second, citizen journalism is not necessarily more liberal, inclusive, or critical of the status quo than traditional media. Although some studies found that social media coverage tends to be favorable of civic action (e.g. Hamdy and Gomaa, 2012; Harlow Johnson, 2011), other studies have also shown that the line between citizen journalism and (mob) vigilantism can be unsettled (Dennis, 2008; Trottier, 2012). Today, nothing captures such concerns better than the meteoric rise of the alt-right (or alternative right) as a label for far right online spaces (the consequences of this for the study of alternative media remain to be further explored). Yet, most of the discussion around citizen journalism and alternative media focuses on cases of activism espousing emancipatory and social justice goals. The literature on the use of social media by ordinary citizens to document the violence and injustice during the Arab Spring or the Gezi Park protests is a case in point. However, such instances of citizen journalism cannot be divorced from their political circumstances (Al-Ghazzi, 2014; Markham, 2014) or from their reliance on mass media in raising awareness of the protests (Ali and Fahmy, 2013).

Therefore, traditional media remain important providers of symbolic meaning of current events for the wider public (Lester and Hutchins, 2009; McChesney and Nichols, 2010; Nielsen and Levy, 2010). Citizen journalists and activists continue to rely upon – and actively seek – the amplification that mass media provide. In addition to that, journalists and mass media institutions have now caught up with the social media logic; the resources at their disposal – from access to politicians to professional footage and the possibility of around the clock synchronous communication – enable them to maintain larger audiences and thus more signifying power than citizen media (Chadwick, 2013: 87, 185). This is also the case in Canada, where newspapers remain a trusted source of information in spite of declining readership rates.^2^ As Canadian audiences embrace social networking sites for their news consumption, so do newspapers and journalists, thus becoming part of the larger ecosystems of content distribution (Canadian Media Research Consortium, 2011). The changes brought about by digital technologies notwithstanding, journalism and news media remain an important – although possibly no longer an exclusive – source of vocabularies, narratives, and judgments (McChesney and Nichols, 2010; Nielsen and Levy, 2010).

### From protest paradigm to the symbolic construction of online engagement

The bodies of research reviewed here suggest that mass media representations continue to be influential in the symbolic construction of protest. That being the case, it would appear that the protest paradigm remains in place. While we agree that it may be premature to declare the demise of the protest paradigm, we argue that it is no longer the only, or the prevalent game in town. New patterns in portraying protest, and civic engagement more generally, have entered the media field in the techno-optimistic climate following the Arab Spring. Our goal in this article is to capture the contours of these emerging new paradigms in the print media’s symbolic construction of civic engagement.

## Method

To map the symbolic construction of engagement advanced by news articles, this article performed a qualitative analysis of relevant stories published in Canadian newspapers between 2005 and 2014. We opted for 2005 as the start date for our search as this is when the popularity of social media reached a critical point and their status as the new paradigm of internet media (web 2.0) was widely recognized. Indeed, we found no articles matching our specifications in 2005 and only one in 2006, confirming our intuition that social media become associated with grassroots engagement in the later period. The corpus of articles (*N* = 423) created for this project is outlined in Table 1.

**Table 1.** Newspaper articles included in the corpus of texts for analysis.

Year	2006	2007	2008	2009	2010	2011	2012	2013	2014	Total
No. of stories selected	1	25	44	46	78	70	54	57	49	423

We used Boolean operators to search the Canadian Newsstand database (which includes all local and national newspapers in Canada) with a combination of keywords: ‘(social media or YouTube or Twitter or Facebook) and (protest* or activis*)’. This combination returned large sets of stories for each year, making it unfeasible to perform a qualitative analysis. Thus, the sample was further narrowed down by

- Excluding articles dealing with engagement in other parts of the world;
- Excluding articles where ‘protest*’ or ‘activis*’ was not linked to grassroots mobilizations using social media;
- Excluding irrelevant use of ‘social media’ and related terms for our goals (e.g. articles where the only reference to social media was the journalist’s Twitter handle).
- Analyzing only the first discussion of a case (in chronological order).

The purposeful exclusion of stories reporting on international cases of engagement (e.g. the Arab Spring or the Occupy movement) remains an important limitation of our study. Freelon et al. (2015) found that such international events have provided journalists with ‘Internet centrist’ discursive resources for making sense of civic mobilizations. During our data collection process, we anecdotally noticed that social media were often taken-for-granted as the tools of revolution against totalitarian regimes and power structures. Since we have excluded these international cases, we cannot say whether or how their coverage shapes the Canadian print’s take on the local cases. Yet, this exclusion had to be performed: the coverage of such cases is a transnational process, and as such part of a larger ecology of communication that exceeded our scope and made our research unfeasible. While larger scale, intensely mediatized events may capture audiences’ imagination, they remain, in a sense, exceptional and outside of citizens’ scope of agency. In contrast, excluding them allowed us to focus on more routine representations of digitally mediated engagement relevant to the readers’ own civic culture. Because our sample is focused on Canada, the stories in it are more likely to contain a more nuanced firsthand reporting rather than retranslation of coverage from international sources. The coverage of international events may have sensitized Canadian journalists and led them to focus on the role of social media in local mobilizations. Nevertheless, their stories represented an attempt to make sense of the new forms of civic mobilization erupting around the globe against the background of Canadian social and political life. In fact, mapping the coverage of Canadian events provides a starting point for further examination of the circulation and mutation of discursive resources across media and geopolitical borders.

Our two-stage analysis focused on who was mobilizing for civic action, for what purposes, against whom, and on the role played by social media in the process. In the first stage, we coded for the initiator of mobilization, the descriptors used for social media, the issue at stake, and the target of the mobilization process. The coding process provided a basis for a quantitative overview of the symbolic construction of engagement. Two of these categories will be discussed in the next section: the initiator or mobilizer and the issue at stake (see Table 2 for an overview of these categories and codes). We developed in vivo codes for issues (i.e. using the terminology proposed by the article), then grouped them by the larger social sector or problem to which they pertained. The codes were not mutually exclusive.

**Table 2.** Categories and codes used in the content analysis stage.

Category	Codes
Initiator or mobilizer of civic engagement through social media	Individual Organization Not mentioned
Issue	Police abuse Public transit International events Budget cuts Formal politics Animal rights Urban development Consumer politics Environment Legal framework Identity (women, LGBT, First Nations) Other Education

LGBT = lesbian, gay, bisexual, and transgender.

In the second stage, articles were reread with an eye to how the initiator was characterized and how social media were presented in relation to engagement. The aim was to chart the discursive contours of technologically mediated engagement, paying attention to recurrent phrases, vocabulary choices, and narrative story lines. This led to three overarching discursive articulations presented next. First, civic mobilization appears as a reaction to already made decisions or existing ideologies. Second, the individual emerges as the driver of this mobilization process. Third, social media become the new social glue, bringing together disconnected individuals into a force of change.

## The (re)active social body

**Figure 1.**
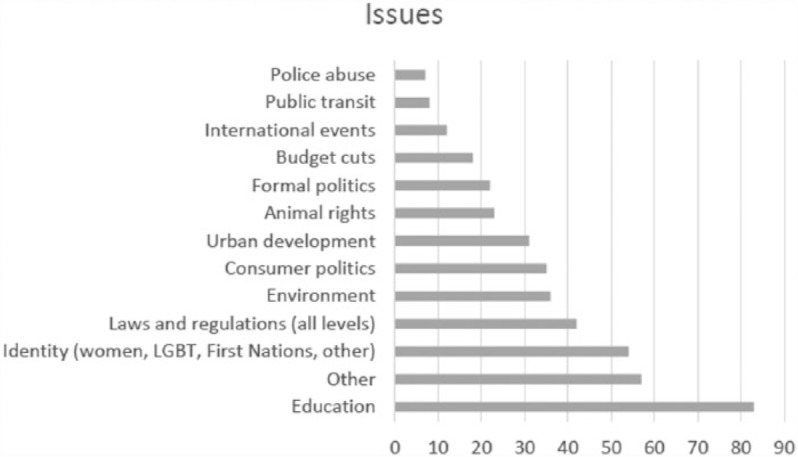
Issue frequency across the sample.

Against current worries of political apathy, the newspaper articles in this sample construct a promising picture of an active social body, with citizens constantly involved in the governance of their lives. When looking at what people mobilized around, the sheer diversity of issues piquing people’s interest is striking (see Figure 1; Table 3 details each code). Citizens mobilized around a range of causes, from reacting to decisions made by local, provincial, or federal governments, corporations, and courts to challenging dominant attitudes and values (e.g. the Earth Hour initiative, asking people to switch off their lights for an hour to raise awareness on climate change; the SlutWalk march aimed at raising awareness on violence against women).

**Table 3.** Breakdown of cases per issue.

Code	Object of civic engagement
Police abuse	Police brutality toward protesters or unarmed citizens
Public transit	Fare increase, scheduling, labor dispute, route cancellations, and changes
International events	International events that trigger local protests or awareness raising actions
Budget cuts	Cuts in the following fields: arts and culture, health, libraries, Coast Guard, social assistance for refugees *Note*: cuts in education were included under Education
Formal politics	Electoral politics, prorogation, public money (excessive spending, allocation including austerity measures), scrapping long census, bilingual signs, opposition to prime minister, social justice demands, opposition to local mayor – Toronto, Québec charter of values
Animal rights	Use of animals in the circus, abuse toward pets (dogs), conditions in petting zoos, pets (dogs) put up for euthanasia for various reasons
Urban development	Closure and demolition of buildings (clubs, bars, historic buildings, schools), urbanization plans (including building new schools, placement of power lines or plants, parks, overpasses, new apartments), urban density, affordable housing
Consumer politics	Demand in services, price hikes, scalping, sale of public utility, closure of various service providers (local TV station, local brewery), support for local producers, fans (firing or hiring of artists, radio hosts, TV anchors, athletes, etc.), commodification of commemoration of war veterans, trademark battle
Environment	Consumer waste, cutting down trees, logging, pipelines, oil and gas industry, mining (coal, precious metals), Earth Hour, parks, genetically modified organisms
Laws and regulations (all levels)	Laws, bills, and regulations on all levels. Includes copyright, natural health products, taxation, Alberta Bill 44 (right of parents to pull out children from controversial topics, including sexual education), ban on beach fires, Bill 82, driving regulations, traffic rules, bylaw regarding street entertainment, surveillance, hunting, legalizing marijuana, public housing regulations, Quebec bylaw 3 – wearing masks, local regulation regarding garbage bags, housing for disabled, Bill 24 – agriculture; Alberta Bill 10 (gay/straight alliances in schools)
Identity (women, LGBT, First Nations, other)	Women: breastfeeding in public, violence toward women; abortion LGBT: pride parades, rights. First Nations: environmental issues affecting First Nations communities (also coded under Environment), food affordability in the North, social justice for First Nations Other: racism, multiculturalism, the Sikh community
Other	Various topics including: award designations, bullying, child abuse, commemoration of historical events, deportations, employment practices, expropriation of farmer, adding fluoride to water, hate groups, hacking collective, court decisions, release of criminals or pedophiles, right of access to beach, prison closure, topic of publicly funded mural, vigilantism/shaming of community members
Education	Food services on campus, recruitment on campus, school dress codes, other school policies, curriculum changes, closure of school or school-related facilities, tuition hikes, cuts to various programs, payment options for tuition, labor disputes, disputes over logo, controversial exhibitions or speakers on campus, teachers/faculty/support staff firing or nonrenewals, grants allocation

LGBT = lesbian, gay, bisexual, and transgender.

The most common targets of grassroots engagement were administrative decisions in the educational sector. Here, issues included everything from changes to campus food service providers and dress codes to tuition hikes and closure of schools or programs (see Table 3). No decision-making level was spared: federal and provincial government, school boards, school principals, university rectors, faculty deans, student or teaching unions, and so on. Citizens also mobilized against harmful practices, as in the case of student-led antibullying campaigns. This may not be surprising though, for the educational sector is prone to both fostering, and being an object of civic engagement (e.g. McKenna and Willms, 1998). Traditionally, the student body has been an active agent of social change. Education is also a unionized sector, which translates into labor disputes that are widely covered by the media. In addition, social media are the preferred tool of the digital generation, so it is no surprise that mobilizations by young people made use of these media more frequently.

The second most common category of civically galvanizing issues was labeled Other. It includes issues that did not fit neatly into the other categories, often pertaining to decisions of governing bodies affecting various areas of social life. Such was the case of ideological mobilizations against the commemoration of certain historical events (e.g. pacifist protests against war-related commemorations) or challenging award designations (e.g. citizen protesting the awarding of a medal to honor a former provincial premier on the grounds of his poor track record in office). In another case, the business improvement authority of a city asked an artist to change her publicly funded mural depicting Karl Marx following complaints from citizens. In turn, other citizens mobilized on behalf of the artist, demanding the protection of artistic freedom. A few of these grassroots initiatives fall under the banner of vigilantism, such as mobilization following the release or presence of criminals or pedophiles in the local communities, or shaming of community members who, although not criminally charged, were deemed ‘guilty’ of unethical acts by other fellow citizens. Ideological disagreement with court decisions also fueled citizen mobilizations.

This diversity of issues portrays an active citizenry relying upon social media to engage with the governance of their lives. Obviously, the reader of a single news story will not necessarily internalize this image. Together, however, the stories in this sample cultivate an understanding of social media grassroots engagement as a commonplace practice. Yet, in portraying engagement as a primarily reactive practice, such stories curtail its scope and function. They place civic engagement at the tail end of decision making as opposed to envisioning possibilities for citizens to become engaged at the earlier stages of the decision-making process and in the very articulation of issues to be deliberated upon.

At the same time, such close-to-home civic engagement typically targets small-scale or trivial issues such as school dress codes, off-piste skiing, hunting permits, the demolition of a strip club, and so on. The democratic ‘worth’ of these cases is ambiguous: Fighting for the right to wear sweatpants is not likely to concern people beyond a particular school setting. Nor is it a matter of social justice or bringing about significant social change. While reminiscent of the slacktivist thesis (Christensen, 2011), questioning the small decisions that affect our lives and doing something about them nevertheless contribute to building the necessary attitudes and skills for an active civic body.

The wide diversity of scenarios of technologically mediated grassroots engagement thus carries a dual potential. It creates a context for trivializing the idea of civic engagement by tying it to narrow utilitarian interests and projects. It places under a common denominator consumerist demands and objections to social injustice, vigilantist pressures, and challenges to structural disparities. At the same time, it highlights the significance of citizens’ ability to talk back to powerful institutions and to stand up together in defense of their interests under different circumstances. Presumably, this experience and its pertaining skills and tactics can travel between contexts and contribute to the growth of a culture in which citizens see themselves as agents, even if not necessarily as activists.

## Power to regular people

The articles in this sample suggest civic engagement is alive and kicking. But they also highlight the crucial driver of engagement: the individual. In the time of social media, the regular person – a cricket fan, an unemployed man, a 19-year-old student, an 85-year-old farmer, a mother, or a local resident – is seemingly unstoppable. This articulation draws its potency from two wider narratives: the celebration of the Internet as empowering people by allowing them to transcend the confines of traditional gender, race, national, or disabled bodies; and, the neoliberal ideology of the free, autonomous individual, in charge of her own life.

**Figure 2.**
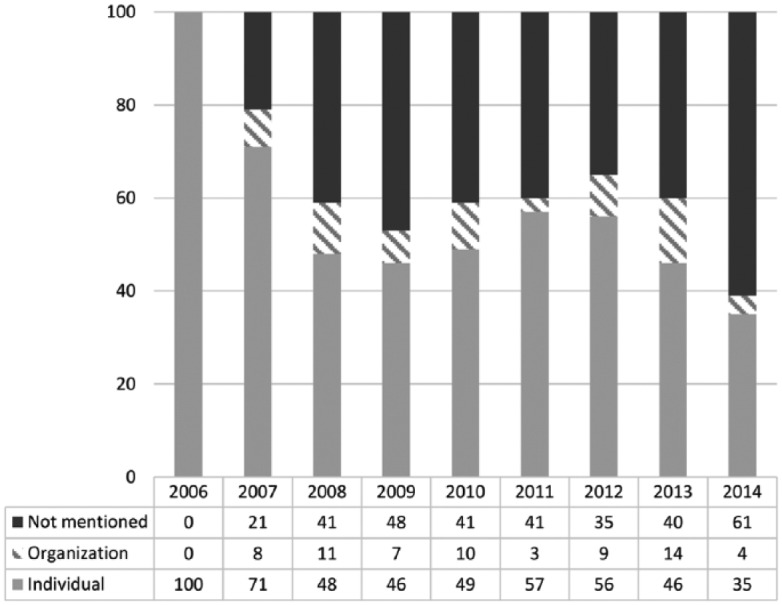
The mobilizer behind the civic engagement cases (percentages).

Take these examples from the sample: Two Grade-12 boys use Facebook and text messaging to mobilize their peers against bullying. In the process, they create a global ‘wear pink’ movement. A 25-year-old goes from ‘animal lover’ to ‘activist’, by relying upon Facebook to advocate for animal rights. The individuals spurring civic action are explicitly named in half of the articles in this sample (Figure 2). They are usually identified by their age, social role, or occupation – all of which construct an image of ‘regular’ people creating ‘exceptional’ outcomes. Since less than 5% of the sample mentions prior activist or political expertise in specific social sectors, the individuals who become engaged do not appear to have any special skills or capital at their disposal. In fact, a lot of them openly express their surprise at the ‘outpouring of support’ that their actions receive. This signals their ‘newness’ to civic mobilization, further inscribing them as ‘regular Janes and Joes’.

If the diversity of issues discussed in the previous section normalizes engagement, the image of the mobilizer outlined above celebrates the power of the individual: anyone, regardless of resources or abilities, can challenge powerful systems. Newswriting practices are conducive to this framing, orienting journalists toward identifying and talking to sources that can offer ‘official’ information or statements on the history and goals of the civic action. When reporting on grassroots movements, journalists may find it difficult to identify ‘spokespersons’; in that respect, social media enabled action brings with it the benefit of being able to contact the person who has set up the Facebook page or Twitter account related to the mobilization. On the other hand, newswriting practices also emphasize the importance of human interest. What makes these stories appealing is precisely the ‘ordinary’ aspect of these exceptional situations, where civic action against institutions, governments, corporations, or prevailing attitudes is undertaken by a seemingly ‘regular’ person. This is, then, the new spin these stories owe to the social media element in the process of engagement: the ordinary person, taken by surprise by the impact of her own action, becomes the star.

Variations in the image of the mobilizer of social-media facilitated civic action are also present across the sample. Occasionally, the initiator of the mobilization process is a group or an organization. Some groups are portrayed as belligerent: The mobilizers are generically described as ‘opponents’, ‘beleaguered citizens’, ‘upset students’, and so on, whose oppositional stance (they are against something) or emotions (they are ‘angry’ or ‘upset’) invokes the specter of danger, an echo of the familiar protest paradigm. Others are more neutrally depicted as teachers, the local public, parents, students, mothers, the arts community, and so on. While such groups may have some expertise (e.g. the arts community can assess the effects of budget cuts upon the development of arts and culture in Canada), this expertise does not qualify them as representatives of an institution with political or administrative power. Thus, these groups are compatible with the image of the regular people as mobilizers. Finally, a small number of articles (see Figure 2) describe the mobilizer as a formal entity (small-scale nonprofit). Nevertheless, in quoting representatives of the organization – for example, a student, an artist, or a mother behind these nonprofits – the engagement scenario becomes represented through an individual.

About half of the sample does not explicitly name the initiator of the civic mobilization process. Such stories either invoke the image of an already existing social media protest group or make passing references to social media as initiators of protest themselves. The mobilizer in these cases is presented as a group of people that has come together via social media. Thus, articles talk of ‘a 25,000-member strong Facebook group’, ‘a thousand students joined a Facebook campaign’, or ‘an online petition so far has 244 signatures’. The numbers convey facticity, fed by journalism’s ‘near-obsession with counting the number of protesters at each event’ (Bishop, 2013: 3). Social media metrics can easily supply reporters with the semblance of objective descriptors of civic mobilization. While such stories do not explicitly mention an individual mobilizer, the numbers invoke the same image of regular people taking part in the civic action.

## The spaces and tools of civic action

The last discursive resource in the newspaper stories focuses on the role of social media in engagement scenarios. Technology appears as the new social glue, bringing isolated individuals together in an efficient and effective manner and transforming them into a force able to pressure decision-making structures.

### Social media: the new sites of civic action

The newspaper articles describe social media as a space where collective action takes place:

The residents … didn’t protest in front of the legislature. They didn’t take over Churchill Square. They did something far more effective. They filled Edmonton’s Twitter stream and blogosphere and Facebook community with intelligent, articulate arguments in favour of their creative, efficient, supportive housing model. (Simons, 2013)

Furthermore, it is online that the individual recognizes herself into others in what amounts to the formation of a community of like-minded people. Once mirrored in others and thus emboldened by the support found online, the individual mobilizer’s first step is to create a Facebook page, set up a Twitter account, start a petition, and so on. ‘I snapped. I had to do something’, declares one such angered individual. ‘I registered a domain name, put up a very simple website, created a Twitter account …’ (Doolittle, 2014).

In such online spaces, ‘people of all stripes are getting together to fight something they don’t like’ (Bundale, 2010). As Facebook groups ‘sprout’ and mass tweet-ins ‘abound’, citizen upon citizen joins in the civic action on social media. Individuals protest, boycott, and pressure decision-making structures online. They sign e-petitions and ‘flood’ politicians with emails, tweets, Facebook posts, or selfies. Sometimes, technologically skilled individuals expose perpetrators, blurring the line between civic action and punishment (such cases, however, are very rarely discussed in the corpus and appear only after 2013, when hackers and Anonymous become visible in the public sphere).

The representation of these new civic spaces implies that social media can easily turn individual engagement into a collective phenomenon. The higher the number of followers or shares, the more impressive the act of engagement and, by extension, the capacity of social networking sites to facilitate the emergence of ‘power in numbers’. Civic engagement may be spurred by one individual, but on social media, it rallies others. Social media thus come to signify a new space of collective, ongoing, and potentially effective civic action.

### Social media: the new tools of engagement

Social media are also tools that individuals can use strategically in becoming and getting others engaged, enabling mobilization or amplification of civic engagement. As tools for organizing or coordinating civic action, these technologies seem to offer cheap and quick alternatives to traditional means of mobilization. As one scholar turned activist explains,

Facebook is more than just a cool way to catch up with old friends; rather, it is an incredibly effective and efficient tool that can be used to educate and galvanize grassroots advocacy, placing unprecedented power into the hands of individuals. (Geist, 2007)

Youth in particular are shown as turning to social media for organizing civic action. When a school district announces changes to spare periods (free time), students ‘launched a Facebook group site to protest … believing that the social networking site would be the most efficient way to gather support for their cause … Facebook is just a great resource for youth. It’s easily accessible from everywhere’ (Lindsay, 2008).

Social media also appear as an amplifier of the individual voice. The individual is now able to ‘put their politics on YouTube’, tell her story on Facebook or express her discontent on Twitter. By making these individual stories visible, ‘new media transform the isolating experiences of individuals into a social phenomenon that is hard to ignore. That in itself is powerful’ (Heartfield, 2011). Thus, social media become the ‘significant extra muscle’ of activism (McMurdy, 2007), partly because of their contagious quality. Thus, issues and causes can trend on Twitter, while Facebook and YouTube protest sites can precipitate a ‘meteoric rise’ in numbers. Occasionally, this contagious dimension can make a difference, resulting in an ‘immediate’ effect or response to mediated engagement.

### Casting doubt upon the civic potential of social media

Not every story in the sample suggests social-media-enhanced activism works. The slacktivist narrative (Christensen, 2011) casts doubt on the worth of Facebook groups or YouTube videos. The few stories in this category report on the disappointment of regular citizens whose attempts at mobilizing others through social media have failed. People are quick to sign a petition or join a group, but that does not necessarily translate in showing up for rallies: ‘People are very fickle online so the challenge is to get them motivated enough to show up in real life’, explains one organizer (Zerbisias, 2011).

Such stories do not focus on specific cases, although they do mention them (which is the reason why they were included in the sample). Instead, they focus on questioning whether clicking or sharing can be seen as either meaningful civic action, or as conducive to it. The answer is ritualistically formulated by juxtaposing the ease of online participation (signing, clicking, posting, etc.) with the difficulty of gathering enough bodies in an offline protest, rally, or sit-in. Individuals are no longer potential mobilizers, but ‘wannabe do-gooders … all about supporting a cause – just as long as they don’t have to get out of their chair’ (Cole, 2011). Lack of effort, saturation with protests and petitions, and inconsequential civic action are all mentioned as potential barriers to social media engagement. In line with standard journalistic practices, such contributions often try to balance the slacktivist charge with a recognition of the potential for engagement. Ultimately, ‘[social media slacktivism] makes communication and dialogue available to those who cannot meet in person … it also connects people in different regions who share a passion for a certain cause’ (Cole, 2011). Even when social media support fails to bring people out, it still initiates the conversation, leaving open the potential for subsequent collective action.

## Discussion

Collectively, the news stories we analyzed advance and popularize a new discursive paradigm of mediated civic engagement characterized by the personalization of engagement, an enthusiasm for the individual as the driver of digital civic action, with an image of social media as a critical tool enabling the participation of citizens in the governance of their polis. Engagement becomes entrenched as a personal decision and action that is amplified – and thus empowered – by social media’s capacity to aggregate it with similar (individual) interests and actions. This paradigm leaves little room for organizations and gives prevalence to online activities over collective action in physical space. The spectacle it foregrounds is that of viral diffusion and spontaneous aggregation online. Such an articulation, we suggest, is part and parcel of the ‘personalization of politics’ (Langer, 2011), consisting of the presidentialization of power (the concentration of power in the hands of individuals) and the personalization of politics (the emphasis on the personal traits and lives of politicians). The emerging news discourse sketched here suggests a new component: the personalization of civic engagement.

In this discourse, civic engagement is symbolically constructed as an individual reaction to already made political and administrative decisions or prevailing norms. The exciting message here is that, thanks to digital mediation, it requires neither skills and resources, nor a collective identity or collective frames for action. The collective may be no longer a premise of civic action because social media ‘rescue’ the individual from an isolated existence and automatically reinsert her into the body politic. The personalized engagement can be smoothly socialized in the spaces and through the tools provided by social media.

The so portrayed personalized engagement appears to be aligned with the ideal of participatory democratic politics. What are, however, its goals and potential achievements? We remain ambivalent on this matter. On one hand, the media discourse suggests social media further the transformation of the personal into the political. The publicity that social media sharing brings to issues and circumstances experienced by the individual as highly personal – for example, not being able to breastfeed in a restaurant, not being able to go out hunting because of new regulations – makes people realize they are not alone. Others are also struggling with these issues – and this realization alone can transform one’s personal hardship and frustration into a consciousness of a social problem paving the way to collective mobilization and action. As such, this discourse opens up an opportunity for individuals to imagine themselves as – and become – active citizens.

Personal struggles or interests do matter, but they are not necessarily compatible with constructing a just and equitable society for everybody. Each case is different – thus distinguishing between the ‘worthy’ and the ‘unworthy’ cases of civic action is a daunting task that we cannot engage with at length here. However, the personalization of engagement carries within it the problem of redefining politics as the incessant pursuit of trivial and selfish private interests. Civic engagement, in other words, is at risk of being understood as the struggle to impose particularistic agendas without the necessity of dialogue, negotiation, and compromise. In the examples provided above, challenging traditional norms associated with breastfeeding that reproduce gendered power relations targets structural inequalities and oppressive cultural norms. Protesting against local hunting regulations, on the other hand, is a grievance of a very different nature (assuming these regulations are not meant to limit access to common resources, impinge on indigenous rights, or create social inequalities and imbalances). Is the self-understanding of citizens as being both entitled and able to stand up and talk back to powerful institutions put in service of the impulse of ‘me first’, or is it driven by an ethic of care for others and an understanding of the complexity of governance in diverse societies? This is a critical question that the new paradigm of portraying social-media fueled civic engagement is yet to incorporate.

## Conclusion

Celebrating an ideal of politics where every individual, through the visibility conferred by social media, can facilitate the aggregation of an interest group to pressure decision-making structures carries both empowering and disempowering repercussions. It is empowering in promoting an image of the average citizen as being able to make a difference in the political process and thus it becomes a source of hope and motivation. It is disempowering in inflating that image beyond structural realities. If engagement is so easy, affordable, and automatically successful, the average individual is equally responsible along with power elites for the persistence of social ills. She has only herself to blame for not standing up to what is wrong in society and fixing it. This is a subtle form of civic responsibilization (Miller and Rose, 2008; Rose, 1999) that places the ordinary citizen under the same denominator with political, administrative, and business institutions and leaders. It creates the false appearance of equality, where citizens allegedly have the same power as elites in affecting decision making. Second, by replacing the notion of the collective with that of the aggregate, this discourse suggests that like-minded people are already out there in the social world and linking up with them would be a painless and unproblematic process (see Bakardjieva, 2015). Playing off from the idea of ‘networked individualism’ (Wellman et al., 2003), it presents the mobilization for a common struggle as a simple and natural click between carriers of already existing common interests. Such a proposition obscures the resources, skills, and painstaking efforts needed to arrive at a shared definition of collective interests and goals among a multitude of individuals in a complex society. Thus, the personalization of digitally mediated engagement presents civic activism as a mere bringing together of pregiven commonalities of interest, belief, and motivation bypassing the difficult, yet formative process of working together with dissimilar others in the name of improving social conditions for all citizens.

## Notes

1. Although we are aware of typologies of engagement that distinguish between political (involvement with formal politics, seeking to influence decision makers and policies) and civic (involvement with local community, in the form of associations, volunteering, charity, etc.) forms (Ekman and Amnå, 2012), we avoid espousing it in this article as, particularly with social media, the boundaries public/private or civic/ political are increasingly untenable (Papacharissi, 2010; Zuckerman, 2015).

2. The NADBank 2015 survey, for instance, found that newspapers reach 70% of Canadians every week, but four out of 10 readers now access them online (News Media Canada, 2015).
